# Complete Genome Sequences of Two Staphylococcus saccharolyticus Strains Isolated from Prosthetic Joint Infections

**DOI:** 10.1128/MRA.00157-21

**Published:** 2021-03-11

**Authors:** Mastaneh Afshar, Anja Poehlein, Bo Söderquist, Holger Brüggemann

**Affiliations:** a Department of Biomedicine, Aarhus University, Aarhus, Denmark; b Department of Genomic and Applied Microbiology, Institute of Microbiology and Genetics, University of Göttingen, Göttingen, Germany; c Department of Laboratory Medicine, Clinical Microbiology, Örebro University Hospital, Örebro, Sweden; University of Maryland School of Medicine

## Abstract

Staphylococcus saccharolyticus is a human skin bacterium and is occasionally associated with prosthetic joint infections (PJIs). Here, we report the complete genome sequences of two strains that were isolated from shoulder and hip PJIs. The genomes show signs of reductive evolution; around 21% of all coding sequences are inactivated by frameshift mutations.

## ANNOUNCEMENT

Staphylococcus saccharolyticus is a coagulase-negative staphylococcal species that is found on normal human skin (1, 2). The species is occasionally associated with infections such as sepsis, prosthetic joint infection (PJI), spondylodiscitis, and other infections (3–6).

Here, we present the complete genome sequences of two S. saccharolyticus strains that were isolated from PJIs in Örebro, Sweden (5). Primary isolation from periprosthetic tissue specimens was achieved by anaerobic incubation in fastidious anaerobic broth (FAB) (2.97% FAB [Lab M, Ltd., Heywood, UK] supplemented with 1% d-glucose) for 5 days, followed by subcultivation on fastidious anaerobic agar (FAA) plates (4.6% FAA [Lab M] supplemented with 5% horse blood). The plates were incubated anaerobically (10% H_2_–10% CO_2_–80% N_2_) at 37°C for 5 days.

High-molecular-weight DNA (HWD) was isolated with the MasterPure complete DNA and RNA purification kit (Biozym Scientific, Hessisch Oldendorf, Germany). The quality of the isolated DNA was initially checked by agarose gel electrophoresis and validated on a Bioanalyzer 2100 (Agilent Technologies, Waldbronn, Germany). The concentration and purity of the isolated DNA were checked with a Nanodrop ND-1000 spectrophotometer (Peqlab Biotechnologie, Erlangen, Germany), and exact concentrations were determined using the Qubit double-stranded DNA (dsDNA) high-sensitivity (HS) assay kit (Life Technologies GmbH, Darmstadt, Germany). Illumina shotgun libraries were prepared using the Nextera XT DNA sample preparation kit and subsequently sequenced on a MiSeq system with the reagent kit v3 with 600 cycles (Illumina, San Diego, CA, USA). Reads were quality filtered using Trimmomatic v0.39 (7), resulting in 2,655,836 (13T0028) and 2,082,444 (strain DVP5-16-4677) paired-end reads. For Nanopore sequencing, 1.5 μg unsheared HWD was used for library preparation with the ligation sequencing kit 1D (SQK-LSK109) and the native barcode expansion kit (EXP-NBD104). Sequencing was performed for 72 h on a MinION Mk1B system with a SpotON flow cell (R9.4.1), using MinKNOW v20.06.4 and Guppy v4.0.9 for base calling (Oxford Nanopore Technologies, Oxford, UK), which resulted in 8,590 (13T0028) and 434,070 (DVP5-16-4677) reads, with *N*_50_ values of 12,541 bp and 1,621 bp, respectively. A hybrid assembly was performed with Unicycler v0.4.6 (8), resulting in circular replicons. Coverage was determined using QualiMap v2.2.1 (9), by mapping Illumina and Nanopore reads on the genomes using Bowtie 2 v2.3.5.1 (10) and minimap2 (11), respectively. The closed genomes have Illumina coverage of 205-fold (13T0028) and 188-fold (DVP5-16-4677) and Nanopore coverage of 10-fold (13T0028) and 153-fold (DVP5-16-4677). Throughout, default parameters were used for all bioinformatic analyses.

The complete genomes consist of chromosomes of 2,332,643 bp (13T0028) and 2,341,587 bp (DVP5-16-4677) and plasmids of 19,923 bp (13T0028) and 55,199 bp (DVP5-16-4677). The GC contents were 32.2% and 32.1% for strains 13T0028 and DVP5-16-4677, respectively. Comparison of strains 13T0028 and DVP5-16-4677 revealed an average nucleotide identity (ANI) of 97.9%, calculated with JSpeciesWS (12). Based on this low ANI, we propose that these strains belong to separate subspecies.

Genome annotation, performed with PGAP (13), predicted 2,221 and 2,215 coding sequences (CDSs) in the genomes of strains 13T0028 and DVP5-16-4677, respectively; large numbers of these, 469 and 493 CDSs, respectively, are not functional due to frameshift mutations or internal stop codons.

### Data availability.

The genome sequences of Staphylococcus saccharolyticus strains 13T0028 and DVP5-16-4677 have been deposited in GenBank under the accession no. CP068029, CP068030, CP068031, and CP068032. The raw reads have been deposited in the NCBI SRA database under the accession no. SRR13570501, SRR13570500, SRR13570499, and SRR13668384.
